# Rupture of interstitial ectopic pregnancy secondary to invasive hydatidiform mole with pelvic metastasis in perimenopausal women: A case report

**DOI:** 10.1097/MD.0000000000048808

**Published:** 2026-05-15

**Authors:** Ling Xu, Yi Li, Tao Wang, Yutai Zhao

**Affiliations:** a Department of Gynaecology and Obstetrics, Ya’an People’s Hospital, Ya’an, Sichuan Province, People’s Republic of China.

**Keywords:** hysterectomy, interstitial ectopic pregnancy, intraperitoneal hemorrhage, invasive hydatidiform mole, pelvic metastases, perimenopause

## Abstract

**Rationale:**

Ectopic gestational trophoblastic disease is an extremely rare subtype of gestational trophoblastic disease, with perimenopausal cases especially scarce and prone to preoperative misdiagnosis as a common ectopic pregnancy, leading to potential delays in proper management.

**Patient concerns:**

A 50-year-old perimenopausal woman (G6P2) presented with acute abdominal pain, dizziness, profuse sweating, and hemorrhagic shock. She had a 4-month amenorrhea, a history of ectopic pregnancy surgery, and uterine curettage. Preoperative assessment suggested ectopic pregnancy rupture with massive intra-abdominal hemorrhage, without initial suspicion of trophoblastic neoplasia.

**Diagnoses:**

Emergency laparoscopic surgery and pathological examination confirmed interstitial ectopic pregnancy rupture secondary to invasive hydatidiform mole with pelvic soft tissue invasion. International Federation of Gynecology and Obstetrics stage II, World Health Organization prognostic score 10 (intermediate-high risk).

**Interventions:**

Emergency laparoscopic wedge resection of the left uterine horn lesion, uterine reconstruction, and bilateral uterine artery ligation were performed. Postoperative standardized etoposide, methotrexate, actinomycin-D, cyclophosphamide, vincristine chemotherapy was administered, followed by 3 cycles of consolidation chemotherapy after beta-human chorionic gonadotropin (β-hCG) normalization.

**Outcomes:**

Serum β-hCG returned to normal at 8 weeks postoperatively. No severe chemotherapy adverse reactions occurred. No recurrence was detected during regular follow-up.

**Lessons:**

Perimenopausal women with amenorrhea, acute abdominal pain, and extremely elevated β-hCG (>10^5^ IU/L) should raise a strong suspicion of ectopic gestational trophoblastic disease. Integrated clinical, imaging, laboratory, and pathological evaluation reduces misdiagnosis. Surgical lesion resection combined with standardized etoposide, methotrexate, actinomycin-D, cyclophosphamide, vincristine chemotherapy, and close follow-up yields a favorable prognosis.

## 1. Introduction

Gestational trophoblastic diseases (GTDs) are a group of diseases resulting from abnormal proliferation of trophoblastic cells in the placenta, and their pathogenesis is associated^[1]^ with a variety of factors such as chromosomal abnormalities in the fertilized egg and maternal immune dysfunction. According to the pathological type and malignancy, GTD can be classified into benign hydatidiform mole and malignant trophoblastic tumors. Among them, invasive hydatidiform mole accounts for 5% to 10% of GTD, mostly secondary to hydatidiform pregnancy, with local invasiveness, which can invade the myometrium and even have distant metastasis.^[2]^ Ectopic pregnancy is a common gynecological acute abdomen, with tubal pregnancy being the most common. In severe cases, it can be life-threatening^[3]^ due to rupture and bleeding.

Ectopic gestational trophoblastic disease (EGTD) refers to trophoblastic lesions occurring outside the uterine cavity. It is extremely rare^[4]^ in clinical practice, with an incidence rate of only 1.5 per 1 million pregnancies, accounting for 0.005% to 0.01% of all ectopic pregnancies and 0.2% to 0.5%^[5]^ of all GTD. Existing studies suggest that partial hydatidiform mole is the most common type of GTD in ectopic sites, accounting for 60% to 70% of ectopic GTD, while invasive hydatidiform mole occurring in ectopic sites is even rarer.^[6]^ In perimenopausal women, ovarian function declines significantly, and the probability of pregnancy is greatly reduced. Cases of EGTD are rarely reported, and due to its clinical manifestations being highly similar to those of common ectopic pregnancy, it is very easy to misdiagnose before surgery, which poses a great challenge^[7]^ to clinical diagnosis and treatment.

This article reports a case of invasive hydatidiform mole with pelvic metastasis secondary to rupture of interstitial ectopic pregnancy in a 50-year-old perimenopausal woman. The clinical features, diagnostic points, and treatment strategies are discussed in combination with literature review, with the aim of improving clinicians’ understanding of this rare disease, reducing misdiagnosis and missed diagnosis, and optimizing the treatment plan.

## 2. Case report

**Figure 1.**
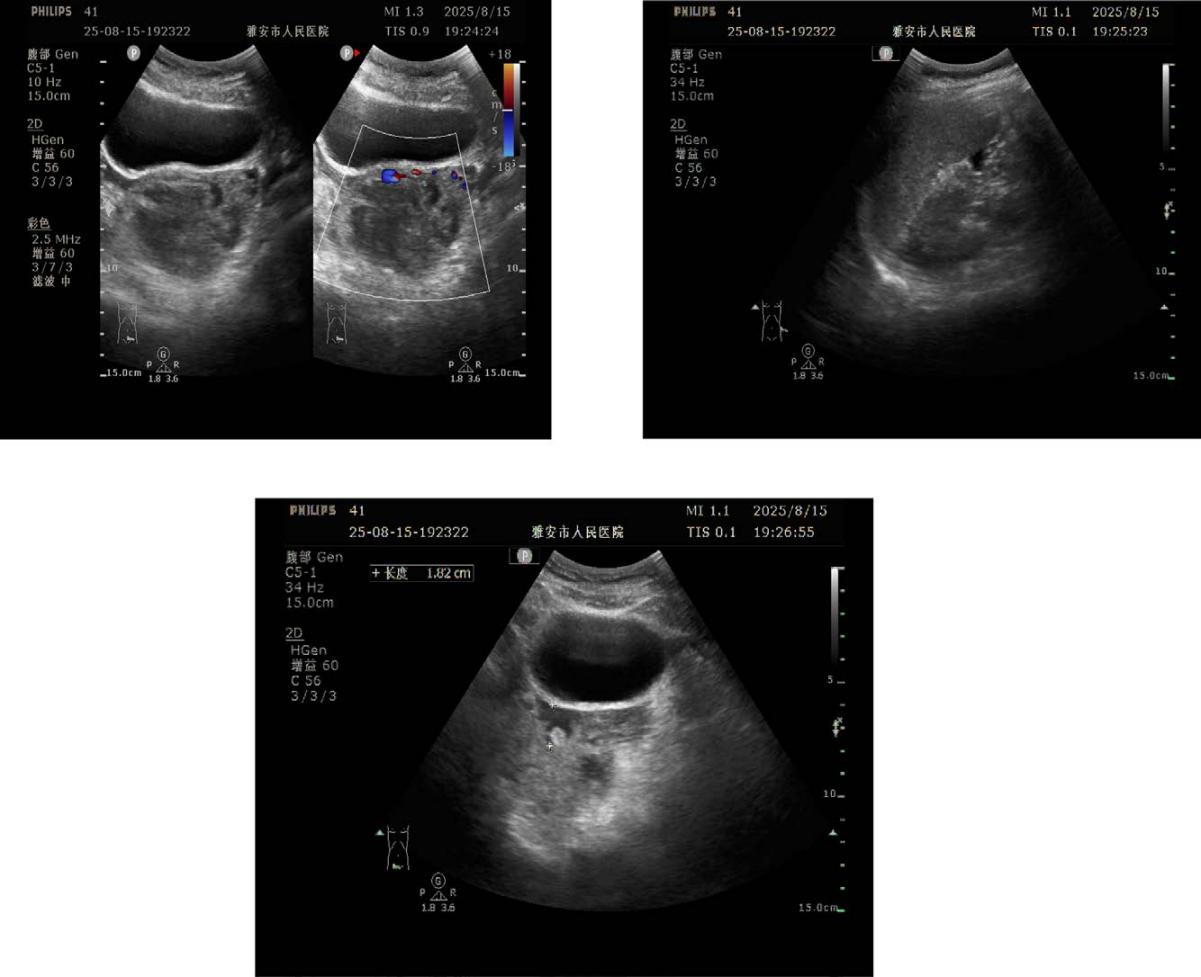
Emergency bedside ultrasound in our hospital showed a normal-sized uterus without a gestational sac in the uterine cavity. A 6.6 cm × 6.3 cm × 5.5 cm mixed-echo mass was detected in the left adnexa, with ill-defined boundaries and irregular shape. CDFI showed abundant blood flow signals around the mass. A large area of hypoechoic region (about 12 cm × 8 cm) was seen in the abdominal cavity, suggesting acute intra-abdominal hemorrhage. CDFI = color Doppler flow imaging.

**Figure 2.**
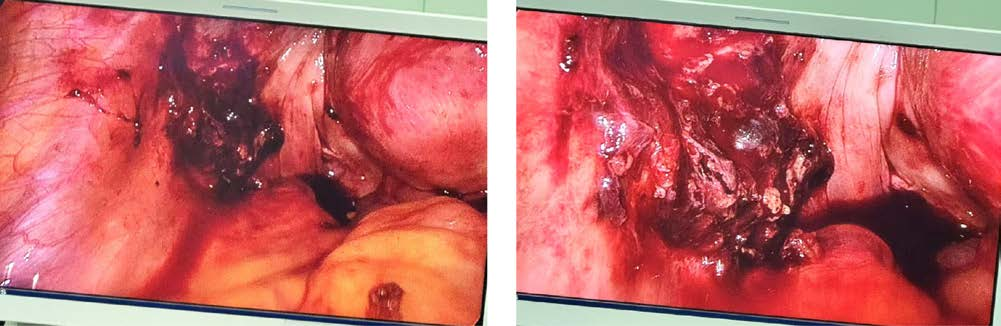
Intraoperatively, a large amount of dark red hemoperitoneum and blood clots were observed, with a total of 1500 mL aspirated. The uterus was normal in size. A 2 cm purple-blue exophytic lesion was noted at the base of the left uterine horn (corresponding to the residual fallopian tube stump) with abundant surface blood vessels. A 6 cm × 5 cm purple-blue lesion was seen in the myometrium adjacent to the left uterine horn, with a 0.5 cm rupture on the surface, accompanied by active bleeding. Obvious vascular proliferation and increased tissue tension were observed in the surrounding myometrium.

**Figure 3.**
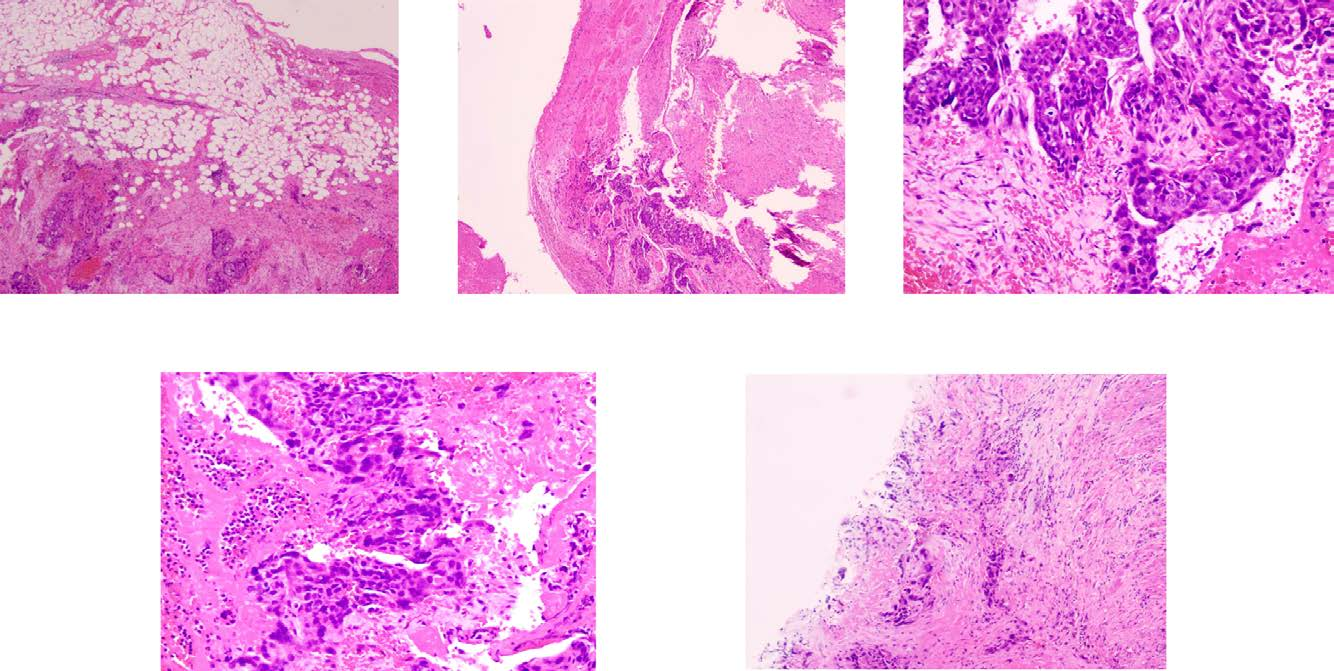
Postoperative routine pathology confirmed invasive hydatidiform mole. Microscopically, villous edema, significant trophoblastic proliferation with obvious cellular atypia were observed. The lesion invaded the myometrium of the left uterine wall to a depth of 1.5 cm, accompanied by rupture and bleeding, and extended extrauterine to the soft tissue of the left pelvic wall.

This study followed the ethical principles of the Declaration of Helsinki, was approved by the Ethics Committee of Ya’an People’s Hospital (approval no. 7622415), and met the requirements of the Case Reporting guidelines. Patients and their families have signed informed consent forms. The patient is a 50-year-old female, 6 years pregnant and 2 giving birth, with no history of drug allergy or long-term medication. She visited the emergency department of our hospital in August 2024 due to “acute abdominal pain for 4 hours, accompanied by dizziness, profuse sweating, and difficulty standing.” The patient had a regular menstrual cycle of 28 to 30 days, a period of 5 to 7 days, and moderate menstrual flow. The patient has been in the transitional period of menopause for the last year, with irregular menstrual cycles, irregular duration and volume of menstruation, and the last menstrual period was in April 2024. Past medical history: a history of regular menstruation was noted in the patient, who is currently in the menopausal transition with approximately 1 year of menstrual irregularities; her last menstrual period was 4 months ago. She underwent unilateral salpingectomy for tubal ectopic pregnancy in 2020, and surgical evacuation for an early intrauterine pregnancy in March 2024, with an uneventful intraoperative course and no abnormal findings. Physical examination: vital signs at admission: blood pressure (141/82 mm Hg), pulse (120 beats per minute), respiration (16 beats per minute), body temperature (36.7°C), blood oxygen saturation (97%). The patient was conscious, listless, pale, with cold and wet limbs, and showed signs of hemorrhagic shock. Abdominal examination: abdominal distension, tenderness throughout the abdomen, rebound tenderness is positive, especially in the lower left abdomen, mobile dullness is positive, and bowel sounds are weakened 2 to 3 times per minute. Gynecological examination: normal vulva development, a small amount of dark red bleeding visible in the vagina, smooth cervix, positive lifting pain, anterior uterus, normal size, medium texture, movable, no obvious tenderness; mild tenderness in the left adnexal area, no obvious mass palpable, and no abnormality palpable in the right adnexal area. Auxiliary examination: bedside emergency ultrasound at our hospital showed a normal size of the uterus and no gestational sac in the uterine cavity. A mixed-echo mass of 6.6 cm × 6.3 cm × 5.5 cm was detected in the left adnexal area, with indistinct boundaries and irregular shapes (Fig. 1). Color Doppler flow imaging showed abundant blood flow signals around the mass. A large area of low echo was seen in the abdominal cavity, about 12 cm × 8 cm, suggesting acute intra-abdominal hemorrhage. Laboratory tests: blood routine: white blood cell count (16.99 × 10^9^/L), neutrophil ratio (85.2%), red blood cell count (2.48 × 10^12^/L), hemoglobin (78 g/L), platelet count (210 × 10^9^/L), suggesting moderate anemia and inflammatory response. Serum beta-human chorionic gonadotropin (β-hCG; 118,584.0 IU/L) was significantly higher than normal pregnancy levels. Coagulation function: prothrombin time 15.60 seconds (reference: 11–13.5 seconds), international normalized ratio 1.47 (reference: 0.8–1.2), fibrinogen 1.92 g/L (reference: 2–4 g/L), D-dimer 2.82 mg/L (reference: <0.5 mg/L). Fibrinogen degradation products 17.20 μg/mL (reference value: <5 μg/mL) suggest abnormal coagulation function, in a hypercoagulable state. No significant abnormalities were observed in liver and kidney function or electrolytes. Positive urine β-hCG test. Preliminary diagnosis: based on the patient’s history of amenorrhea, symptoms of acute abdominal pain, signs of hemorrhagic shock, and results of auxiliary examinations, the preliminary diagnosis is hemorrhagic shock; rupture of ectopic pregnancy. Surgical treatment: laparoscopic exploration was performed in an emergency as the patient’s condition was critical. During the operation, a large amount of dark red accumulated blood and clots were seen in the abdominal cavity. A total of 1500 mL of accumulated blood was aspirated using an aspirator. The uterine size was normal. A 2 cm purple-blue exophytic lesion was observed in the base of the left uterine horn, with abundant surface blood vessels corresponding to the position of the residual fallopian tube stump. A 6 cm × 5 cm purple-blue lesion was seen in the myometrium adjacent to the left uterine horn. There was a 0.5 cm rupture on the surface of the lesion with active bleeding. The myometrium around the lesion showed obvious vascular hyperplasia and increased tissue tension. No abnormalities were found in the ovaries on both sides, but the right fallopian tube was absent (Fig. 2). A wedge resection of the left lesion uterus + uterine reconstruction + bilateral uterine artery ligation was performed. Complete resection of the lesion tissue during the operation, multilayer uterine reconstruction using continuous locking suture technique, and bilateral uterine artery ligation to reduce the risk of postoperative bleeding. Four units of suspended red blood cells and 4 units of fresh frozen plasma were transfused during the operation to correct anemia and abnormal coagulation function. The operation went smoothly. Pathological diagnosis: rapid intraoperative pathology suggests that the surface of the lesion tissue is covered with intact serosa, beneath which a large number of grayish-white, highly branched reticular structures are visible, and trophoblast hyperplasia is obvious, suggesting hydatidiform mole with invasive growth. Postoperative routine pathology confirmed invasive hydatidiform mole, microscopic villous edema, significant proliferation of trophoblast cells, obvious cell atypia, infiltration to a depth of 1.5 cm in the myometrium of the left uterine wall, accompanied by rupture and bleeding, and extrauterine extension to the soft tissue of the left pelvic wall. Immunohistochemical results: HCG (+), Ki-67 (proliferation index about 70%), p53 (scattered +; Fig. 3). Staging and scoring: postoperatively, complete computed tomography scans of the chest, abdomen, pelvis, and brain showed no distant metastases in the lungs, liver, brain, etc. According to the 2000 International Federation of Gynecology and Obstetrics gestational trophoblastic tumor staging criteria, it was diagnosed as stage II invasive hydatidiform mole; combined with the patient’s age (50 years), serum β-hCG level (>10^5^ IU/L), disease duration (<4 months), maximum tumor diameter (6 cm), and metastasis site (pelvic), the World Health Organization (WHO) prognostic score was 10 points, belonging to the medium-high risk group. Postoperative treatment and follow-up: on the seventh day after the operation, the patient recovered well and began chemotherapy with the etoposide, methotrexate, actinomycin-D, cyclophosphamide, vincristine (EMA-CO) regimen, which was intravenous infusion of etoposide 100 mg/m^2^ on the first and second days; methotrexate 100 mg/m^2^ intravenous injection, day 1, methotrexate 200 mg/m^2^ intravenous infusion for 12 hours, day 1; actinomycin-D 0.5 mg intravenous injection, day 1 to 2; cyclophosphamide 600 mg/m^2^ intravenous drip, day 8; vincristine 1 mg/m^2^ intravenous injection, day 8. During chemotherapy, symptomatic and supportive treatments such as antiemetic, liver-protecting, and leukocyte-raising were given. No serious adverse reactions occurred in the patient, only mild nausea and alopecia, which were tolerable. Serum β-hCG levels were monitored weekly, and 8 weeks after surgery, to the normal reference range (<5 IU/L). Three cycles of consolidation chemotherapy were continued. Regular follow-up was conducted after the end of chemotherapy to monitor serum β-hCG and gynecological ultrasound. No clinical symptoms or abnormal laboratory indicators were found so far, and no signs of disease recurrence were observed.

## 3. Discussion

This case has multiple rare clinical features that are extremely rare in clinical practice and have significant academic reference value. First, the age of onset is special. The patient is a 50-year-old perimenopausal woman. Women in this age group have decreased ovarian function, fewer follicles, a lower frequency of ovulation, a significantly lower probability of natural pregnancy, and a much lower risk of GTD than women^[8]^ of childbearing age. Less than 20 cases have been reported globally, and this case further enriches the clinical data of EGTD in this age group.

Secondly, the lesion was located in the uterine stroma rather than in the fallopian tube, which is the most common site^[9]^ for EGTD. Interstitial pregnancy refers to the implantation of the fertilized egg in the myometrium at the junction of the uterine horn and the opening of the fallopian tube. It is a special type of ectopic pregnancy, with an incidence rate of <5%^[10]^ in ectopic pregnancies, and the combination of invasive hydatidiform mole is even rarer, with <10 cases reported in domestic and foreign literature at present, and the mortality rate of extrauterine metastasis is relatively^[11]^ high. The muscular layer of the interstitial part is thick and rich in blood supply. The early symptoms of the lesion are atypical. Once it ruptured and bled, the amount of bleeding is often large, which can easily lead to hemorrhagic shock and endanger the patient’s life.^[12,13]^

In this case, hemorrhagic shock occurred due to the rupture of the interstitial lesion, and the condition was extremely dangerous. In addition, the patient has a history of 1 ectopic pregnancy surgery and 1 curettage. Multiple pelvic and uterine operations may lead to pelvic adhesions and endometrial damage, affecting the normal transport and implantation of the fertilized egg and increasing the risk^[14]^ of ectopic pregnancy. At the same time, multiple pregnancy-related operations may lead to residual trophoblast cells, providing conditions^[15]^ for abnormal proliferation and malignant transformation of trophoblast cells, which may be an important contributing factor to invasive hydatidiform mole in this case.

In terms of pathogenesis, perimenopausal women experience significant fluctuations in hormone levels, with decreased levels of estrogen and progesterone, which may affect the normal differentiation and apoptosis of trophoblast cells and lead to abnormal proliferation.^[16]^ At the same time, after right unilateral salpingectomy, the transport of the fertilized egg is blocked, and it may implant in the left interstitial part of the uterus, resulting in ectopic pregnancy. Subsequently, the trophoblast cells further undergo malignant transformation and develop into an invasive hydatidiform mole, which invades the muscular layer and pelvic lateral wall, eventually leading to rupture and bleeding.

Clinical diagnostic difficulties and differential diagnosis: this case was initially diagnosed as a common ectopic pregnancy before the operation, and was confirmed as an invasive hydatidiform mole after the operation through pathological examination. This reflects the difficulties in the clinical diagnosis of EGTD. The main reasons include the following aspects: first, the clinical manifestations lack specificity. The symptoms of EGTD patients are highly similar to those of common ectopic pregnancy, mostly presenting as postmenopausal abdominal pain, vaginal bleeding, and hemorrhagic shock in severe cases, while the typical symptoms of GTD, such as hyperemesis gravidarum, preeclampsia, and hyperthyroidism, are less common in ectopic lesions, making it difficult for clinicians to differentiate through symptoms. In this case, the patient only presented with symptoms such as acute abdominal pain, dizziness, and profuse sweating, without the specific manifestations of GTD, which increased the difficulty of preoperative diagnosis. Secondly, the imaging examination was limited. The preoperative ultrasound in this case only indicated a mixed-echo mass in the left adnexal area and intra-abdominal hemorrhage, and no specific imaging features of hydatidiform mole were found, which could not provide a direct basis for the diagnosis of EGTD. In addition, clinicians are less vigilant about the occurrence of EGTD in perimenopausal women and tend to overlook key differential diagnostic indicators. Serum β-hCG levels are an important basis for differentiating common ectopic pregnancy from EGTD. Serum β-hCG levels in patients with common ectopic pregnancy are usually <10^4^ IU/L,^[17]^ while in patients with EGTD, due to abnormal proliferation of trophoblast cells, β-hCG levels are significantly elevated, often >10^5^ IU/L. In this case, the patient’s serum β-hCG level was as high as 118,584.0 IU/L, which was much higher than the level of normal ectopic pregnancy, but it was not given sufficient attention before the operation, resulting in misdiagnosis. The differential diagnosis of EGTD mainly includes the following diseases: common ectopic pregnancy; intrauterine invasive hydatidiform mole; choriocarcinoma; and ovarian tumor.

The selection and rationality of the treatment plan: this case adopted a comprehensive treatment plan combining surgery and chemotherapy, achieving good therapeutic effects. The selection of the treatment plan conforms to clinical guidelines and the individualized situation of the patient, and has important reference significance. The primary purpose of emergency laparoscopic surgery was to stop the bleeding, save the patient’s life, and completely remove the lesion tissue to lay the foundation for subsequent chemotherapy. Considering that the patient was in the perimenopausal period but did not explicitly give up fertility, and the lesion was confined to the left uterine horn, a wedge resection of the uterine horn was chosen instead of a total hysterectomy, which completely removed the lesion tissue and maximally preserved the patient’s fertility and the anatomical structure of the uterus, in line with the principle of individualized treatment. Bilateral uterine artery ligation was performed during the operation, which further reduced the risk of postoperative bleeding and improved the safety of the surgery. Laparoscopic surgery, which has the advantages of small trauma and quick postoperative recovery, is increasingly widely used in emergency situations. The operation in this case went smoothly, and the patient recovered well after surgery, demonstrating the advantages of laparoscopic surgery in the treatment of EGTD. The choice of chemotherapy regimen was based on the patient’s International Federation of Gynecology and Obstetrics stage and WHO prognostic score. This case was a stage II invasive hydatidiform mole, with a WHO prognostic score of 10, belonging to the medium-high risk group. The EMA-CO regimen is currently internationally recognized as the first-line chemotherapy regimen for medium-high risk gestational trophoblastic tumors, with a total effective rate of over 90%. And the adverse reactions are relatively manageable. The choice of postoperative consolidation chemotherapy is also in line with the clinical guideline recommendations. For patients with medium-high-risk invasive hydatidiform mole, consolidation chemotherapy should be given for 2 to 3 cycles after serum β-hCG returns to normal to reduce the risk of disease recurrence. In this case, after the β-hCG returned to normal, 3 cycles of EMA-CO regimen consolidation chemotherapy were continued, and no recurrence was observed during the follow-up to date, indicating the necessity of consolidation chemotherapy. During chemotherapy, symptomatic and supportive treatment was given, which effectively reduced the adverse reactions of chemotherapy and improved patient tolerance.

## 4. Conclusion

In conclusion, interstitial ectopic pregnancy rupture secondary to invasive hydatidiform mole with pelvic metastasis in perimenopausal women is an extremely rare and clinically challenging condition. It is easily misdiagnosed preoperatively as common ectopic pregnancy due to atypical clinical manifestations and lack of specific imaging features. Extremely high serum β‑hCG level is an important warning indicator for this disease. Timely emergency surgery to control bleeding and resect lesions, combined with postoperative standardized EMA‑CO chemotherapy and close long‑term follow‑up, can effectively improve the prognosis and reduce the risk of recurrence. Clinicians should strengthen the awareness of this rare disease, integrate clinical, laboratory, imaging and pathological findings for comprehensive evaluation, so as to avoid misdiagnosis and missed diagnosis, and optimize individualized treatment strategies.

## Author contributions

**Conceptualization:** Ling Xu, Yi Li, Tao Wang, Yutai Zhao.

**Data curation:** Ling Xu, Yi Li.

**Writing – original draft:** Ling Xu, Yutai Zhao.

**Writing – review & editing:** Ling Xu, Yi Li, Tao Wang, Yutai Zhao.
